# Pelvic Floor Adaptation to a Prenatal Exercise Program: Does It Affect Labor Outcomes or Levator Ani Muscle Injury? A Randomized Controlled Trial

**DOI:** 10.3390/diagnostics15151853

**Published:** 2025-07-23

**Authors:** Aránzazu Martín-Arias, Irene Fernández-Buhigas, Daniel Martínez-Campo, Adriana Aquise Pino, Valeria Rolle, Miguel Sánchez-Polan, Cristina Silva-Jose, Maria M. Gil, Belén Santacruz

**Affiliations:** 1 Department of Obstetrics and Gynecology, Hospital Universitario de Torrejón, Torrejón de Ardoz, 28850 Madrid, Spain; ifernandezb@salud.madrid.org (I.F.-B.); dmcampo@torrejonsalud.com (D.M.-C.); aaquise@torrejonsalud.com (A.A.P.); bsantacruz@torrejonsalud.com (B.S.); 2 Faculty of Medicine, Universidad Francisco de Vitoria, Pozuelo de Alarcón, 28223 Madrid, Spain; 3 Spanish Network in Maternal, Neonatal, Child and Developmental Health Research (RICORS-SAMID, RD24/0013/0018) Instituto de Salud Carlos III, 28040 Madrid, Spain; avleria77@gmail.com; 4 Plataforma de Bioestadística y Epidemiología, Instituto de Investigación Sanitaria del Principado de Asturias, Avenida Hospital Universitario s/n, 33011 Oviedo, Spain; 5 Statistics Department, Fundación para la Investigación y el Desarrollo de la Medicina Materno-Fetal y Neonatal, iMaterna, Alcalá de Henares, 28806 Madrid, Spain; 6 GICAF Research Group, Department of Education, Research and Evaluation Methods, Universidad Pontificia Comillas, 28049 Madrid, Spain; 7 AFIPE Research Group, Faculty of Physical Activity and Sport Sciences-INEF, Universidad Politécnica de Madrid, 28040 Madrid, Spain; cristina.silva.jose@upm.es; 8 Department of Obstetrics and Gynecology, Hospital Universitario La Paz, 28261 Madrid, Spain

**Keywords:** pregnancy, exercise, pelvic floor ultrasound, delivery, levator ani avulsion

## Abstract

**Background:** Physical exercise during pregnancy is strongly recommended due to its well-established benefits for both mother and child. However, its impact on the pelvic floor remains insufficiently studied. This study aimed to evaluate pelvic floor adaptations to a structured prenatal exercise program using transperineal ultrasound, and to assess associations with the duration of the second stage of labor and mode of delivery. **Methods:** This is a planned secondary analysis of a randomized controlled clinical trial (RCT) (NCT04563065) including women with singleton pregnancies at 12–14 weeks of gestation. Participants were randomized to either an exercise group, which followed a supervised physical exercise program three times per week, or a control group, which received standard antenatal care. Transperineal ultrasound was used at the second trimester of pregnancy and six months postpartum to measure urogenital hiatus dimensions at rest, during maximal pelvic floor contraction, and during the Valsalva maneuver, to calculate hiatal contractility and distensibility and to evaluate levator ani muscle insertion. Regression analyses were performed to assess the relationship between urogenital hiatus measurements and both duration of the second stage of labor and mode of delivery. **Results:** A total of 78 participants were included in the final analysis: 41 in the control group and 37 in the exercise group. The anteroposterior diameter of the urogenital hiatus at rest was significantly smaller in the exercise group compared to controls (4.60 mm [SD 0.62] vs. 4.91 mm [SD 0.76]; *p* = 0.049). No other statistically significant differences were observed in static measurements. However, contractility was significantly reduced in the exercise group for both the latero-lateral diameter (8.54% vs. 4.04%; *p* = 0.012) and hiatus area (20.15% vs. 12.55%; *p* = 0.020). Distensibility was similar between groups. There were no significant differences in the duration of the second stage of labor or mode of delivery. Six months after delivery, there was an absolute risk reduction of 32.5% of levator ani muscle avulsion in the exercise group compared to the control group (53.3% and 20.8%, respectively; *p* = 0.009). **Conclusions:** A supervised exercise program during pregnancy appears to modify pelvic floor morphology and function, reducing the incidence of levator ani muscle avulsion without affecting the type or duration of delivery. These findings support the safety and potential protective role of prenatal exercise in maintaining pelvic floor integrity.

## 1. Introduction

Regular and supervised exercise during pregnancy is widely recommended due to its well-established benefits for maternal and fetal health [1]. Evidence suggests that prenatal exercise may help prevent or manage pregnancy-related complications such as preeclampsia, gestational diabetes, and excessive maternal weight gain [1,2,3,4,5,6,7,8,9,10,11,12,13]. However, the effects of exercise on the maternal pelvic floor remain less clear. In 2004, two contradictory hypotheses were proposed regarding the impact of exercise on the women’s pelvic floor [14]. According to the first, repeated mechanical stress during physical activity induces reflex pelvic floor contractions, potentially strengthening these muscles through an indirect training effect. This adaptation could lead to hypertrophy and shortening of the pelvic floor musculature, reducing the area of the urogenital hiatus and potentially lowering the risk of pelvic floor disorders such as urinary incontinence and prolapse. However, a more rigid pelvic floor may also hinder fetal descent during childbirth. Conversely, the second hypothesis posits that increased intra-abdominal pressure during physical activity may overload and weaken the pelvic floor, leading to widening of the urogenital hiatus. While this could facilitate vaginal delivery by reducing resistance during fetal descent, it may also increase the long-term risk of incontinence and prolapse.

Current evidence remains inconclusive. While some studies indicate that prenatal exercise is associated with shorter labor and lower rates of cesarean delivery [15], others find no significant influence on labor duration or delivery mode [16,17]. In this context, transperineal ultrasound has emerged as a valuable, non-invasive tool for assessing pelvic floor morphology and function [18], offering insights into how exercise might influence labor outcomes. By measuring structures such as the urogenital hiatus, this imaging technique can help establish correlations between pelvic floor adaptations and labor outcomes [19,20,21].

This study aims to evaluate the effects of a supervised physical exercise program during pregnancy on the pelvic floor morphology and function as well as its potential associations with the duration of the second stage of labor, mode of delivery, and pelvic floor injury six months postpartum. A better understanding of these relationships could inform evidence-based recommendations for prenatal exercise and improve maternal care.

## 2. Materials and Methods

### 2.1. Trial Design and Participants

This is a planned secondary analysis of a multicentric randomized controlled trial (RCT) (NCT04563065). In brief, this was a multicentric clinical study conducted via a collaboration between the Obstetrics and Gynecology Department of four maternity units in Spain and the Universidad Politécnica de Madrid. For this secondary analysis, only data from Hospital Universitario de Torrejón were used.

All consecutive pregnant women attending their first-trimester hospital appointment were invited to participate. Eligibility criteria were singleton pregnancy, absence of obstetric complications as defined by the American College of Obstetricians and Gynecologists (ACOG) guidelines [22], gestational age at recruitment less than 14^+3^ weeks, no participation in other supervised exercise programs during pregnancy, and the ability to communicate in Spanish.

Trial coordinators conducted regular quality control checks to ensure protocol adherence and data integrity. This study was approved by the Local Research Ethics Committee (CEIM Hospital Universitario Elche-Vinalopó) and all women provided written informed consent.

### 2.2. Randomization

Randomization was performed between 12^+0^ to 14^+3^ weeks of gestation using a computer-generated block randomization sequence with a 1:1 allocation ratio, The sequence was uploaded into the REDCap software [23] to allocate participants to either the exercise group or control group.

### 2.3. Intervention Program

Pregnant women assigned to the exercise group followed a supervised virtual exercise program throughout pregnancy, starting between 12 and 14 weeks of gestation. The program followed the Barakat model, developed by our research group, with seven parts: 5 min warm-up, 20 min aerobic exercises, 10 min strength training, 10 min balance and coordination exercises, 7 min pelvic floor muscle training (slow and fast Kegel Contractions coordinated with breath), 5 min stretching and relaxation, and 3 min post-session communication [24]. It included three 55–60 min sessions per week and consisted of: (a) one session of individual work using pre-recorded videos hosted on a private YouTube playlist and designed with indications and visual information for easy following; and (b) two live, group supervised sessions conducted via Zoom, scheduled on separate days to accommodate participants’ availability.

Adherence was defined as attendance at ≥80% of the total sessions during the gestational period, calculated as the number of attended sessions divided by the total number of planned sessions [25].

### 2.4. Control Group

Pregnant women allocated to the control group were advised to continue with their usual daily activities but not to participate in any structured exercise programs exceeding 30 min per session, three times per week. Physical activity volume was assessed at a final interview between 38^+0^ and 39^+6^ weeks of gestation by an exercise specialist.

### 2.5. Follow-Up

Both groups received the same prenatal care at the Hospital Universitario de Torrejón. Obstetric appointments took place at 12^+0^–13^+5^, 19^+0^–21^+6^, 27^+0^–28^+6^ and 35^+0^–36^+6^ weeks of gestation, with an additional postpartum visit 6–12 months after delivery.

Maternal characteristics and history and baseline measurements were recorded during the first visit at 12^+0^ to 13^+5^ weeks. These included maternal age, height, weight, pre-pregnancy body mass index (BMI), parity (defined as previous delivery ≥ 24 weeks), method of conception (spontaneous or assisted), smoking status before pregnancy, and pre-pregnancy physical activity level [no activity, occasional exercise but not regular, active (twice per week), very active (3–4 times per week), athlete (daily exercise)]. Maternal blood pressure and maternal weight were recorded at each visit.

### 2.6. Pelvic Floor Ultrasound

**Figure 1:**
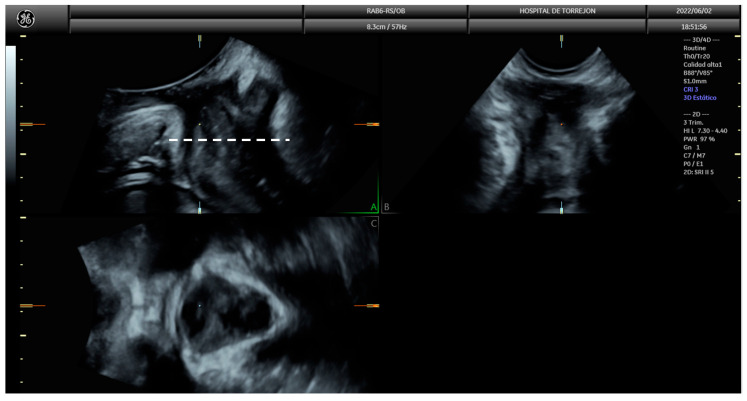
Multiplanar view of translabial pelvic floor ultrasound. Plane (**A**) displays the mid-sagittal plane acquired directly during the scan. The dashed white line indicates the location of the axial plane used for measuring the hiatal diameters and area. Plane (**B**) shows coronal plane and plane (**C**) shows the 3D reconstruction of the axial view of the urogenital hiatus, where hiatal dimensions are assessed.

**Figure 2:**
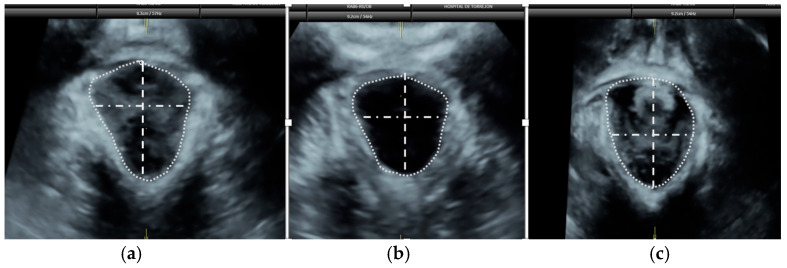
Urogenital hiatus diameters and area from the same patient at 20 weeks of gestation, acquired at rest (**a**), during maximal levator ani contraction (**b**), and during the Valsalva maneuver (**c**). Dashed lines indicate the anteroposterior diameter, measured from the inferior border of the symphysis pubis to the medial margin of the levator ani muscle. Dash-dotted lines represent the transverse diameter, defined as the widest distance between the medial borders of the levator ani muscle, perpendicular to the anteroposterior diameter. Dotted lines delineate the levator hiatus area, bounded by the symphysis pubis, inferior pubic ramus, and medial margins of the levator ani muscle.

Pelvic floor ultrasound assessment was performed at 19^+0^ to 21^+6^ weeks of gestation and 6–12 months after delivery, using a Voluson S10 Expert ultrasound machine (GE Healthcare; Zipf, Vöcklabruck, Austria) with a three-dimensional (3D) convex transducer (RAB6-RS, GE Healthcare; Zipf, Austria). Scans were performed with an empty bladder in the midsagittal plane, and six 3D volumes were acquired per patient: two at rest, two during maximal pelvic floor contraction, and two during the Valsalva maneuver. Prior to image acquisition, participants received standardized instructions on performing both maneuvers. Offline image analysis was performed using the 4D View^®^ (GE Healthcare; Zipf, Austria) software, three months after delivery, by two investigators blinded to delivery outcomes. For each volume, anterior–posterior and latero-lateral diameters of the urogenital hiatus, as well as hiatus area, were measured following the method of Dietz et al. [26] (Figure 1 and Figure 2).

Contractility was defined as the proportional change from rest to maximum contraction using the formula [27]:

100 × [(measurement_rest_ − measurement_contraction_)/measurement_rest_)]

Distensibility was defined as the proportional change during the Valsalva maneuver [27] and calculated as:

100 × [(measurement_Valsalva_ − measurement_rest_)/measurement_rest_)].

**Figure 3:**
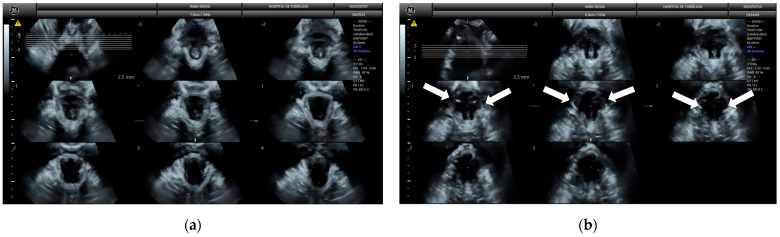
Tomographic ultrasound imaging (TUI) of normal levator ani muscle insertion (**a**) and complete bilateral avulsion of the levator ani muscle (**b**), demonstrated by absence of visible muscle attachment at the pubic bone across the central slices (white arrows).

Puborectalis muscle avulsion was assessed using tomographic ultrasound imaging (TUI) with 2.5 mm slice intervals on volumes obtained during pelvic floor muscle contraction (Figure 3). Complete levator avulsion was diagnosed when abnormal muscle insertion was observed in the reference slice and in the slices located 2.5 mm and 5.0 mm cranial to it [28].

### 2.7. Statistical Analysis

Continuous variables were described as mean (standard deviation), and categorical variables as n (%). Between-group comparisons were conducted using Fisher’s exact test or the Mann–Whitney U, as appropriate. To assess differences in labor outcomes, logistic regression (for mode of delivery) and linear regression (for second-stage duration) were used. Each pelvic floor measurement was analyzed in a separate model, adjusted for maternal age, BMI and parity. All analyses were performed using the statistical software R (version 4.4.2) [29].

### 2.8. Sample Size Calculation

Sample size calculation for the original RCT was reported somewhere else [30].

## 3. Results

**Figure 4:**
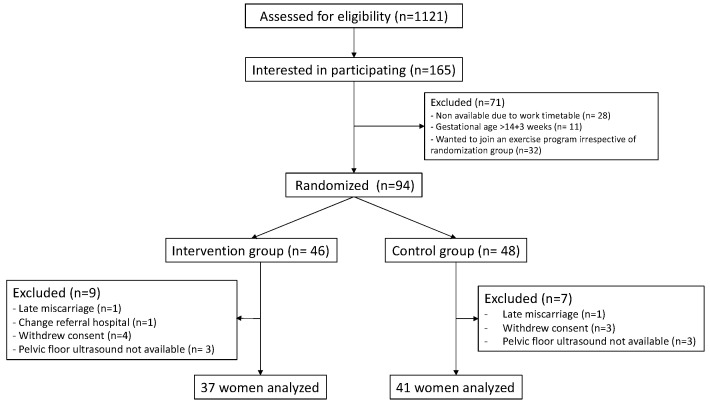
Patients flow diagram.

Between March 2021 and March 2022, 94 pregnant women were recruited; 48 women were randomized to the control group and 46 to the exercise group. After appropriate exclusions, 78 participants were included in the analysis, 41 in the control group and 37 in the exercise group (Figure 4). Maternal and pregnancy characteristics of the study population are shown in Table 1.

Pelvic floor ultrasound findings from the second trimester and postpartum period are presented in Table 2. The antero-posterior diameter of the urogenital hiatus at rest was significantly smaller in the exercise group compared to the control group (4.91 [SD 0.76] mm vs. 4.60 [SD 0.62] mm; *p* = 0.049). No other statistically significant differences were found in static pelvic floor measurements between the groups. When evaluating contractility (the proportional change from rest to maximal contraction), women in the control group showed greater changes in the latero-lateral hiatus diameter (8.54% vs. 4.04%; *p* = 0.012) and hiatus area (20.15% vs. 12.55%; *p* = 0.020) compared to the exercise group (Table 2). There were no statistically significant differences in distensibility (change during Valsalva) between the groups nor in mode of delivery (spontaneous vs. instrumental), and pelvic floor measurements were not associated with delivery type (Table 3 and Table S1).

Similarly, no differences were found between groups in the duration of the second stage of labor. Ultrasound measurements, including urogenital hiatus dimensions and Valsalva-induced distension, were not associated with the length of this stage (Table S2).

Pelvic floor ultrasound was performed at six months postpartum in a total of 54 patients, 30 from the control group and 24 from the exercise group. No statistically significant differences were found in the measurements of the urogenital hiatus (Table 4). However, a lower incidence of levator ani muscle avulsion was observed in the exercise group (5 of 24, 20.8%) compared to the control group (16 of 30, 53.3%; *p* = 0.009), with an absolute risk reduction of 32,5%. Additionally, greater distensibility was observed in the control group compared to the exercise group, both in the lateral diameter and the area of the hiatus (Table 4).

## 4. Discussion

### 4.1. Main Findings

The main findings of this study are the following: first, women who participated in the supervised exercise program had a smaller anteroposterior diameter at rest compared to those who did not; however, contractility was lower in the exercise group; second, the type of delivery and the duration of the second stage of labor did not differ significantly between both groups; third, second trimester pelvic floor measurements were not associated with the duration of the second stage of labor or the mode of delivery; and fourth, women in the exercise group had a lower incidence of levator ani muscle avulsion and reduced hiatal distensibility compared to controls.

### 4.2. Comparison with Previous Studies

Few studies have examined the effects of an exercise program during pregnancy on the pelvic floor muscles. In 2015, Bø et al. reported that primiparous women who exercised regularly had a larger hiatus area than those who did not exercise, with no differences observed in the type or duration of labor [18]. In contrast, our study found a smaller anteroposterior diameter at rest and lower contractility in the exercise group. The smaller anteroposterior diameter of the urogenital hiatus at rest and lower contractility in women who exercised during pregnancy may reflect an increased pelvic floor muscle tone, resulting in reduced elasticity and contractile response, but also muscle fatigue or neuromuscular coordination alterations. Future research should analyze training intensity, muscle fatigue, and pelvic floor activation patterns.

Regarding labor outcomes, our findings did not support an association between pelvic floor measurements and the duration of labor. Recently, several studies have explored the influence of pelvic floor muscles on childbirth outcomes. Lanzarone et al. conducted a prospective observational pilot study in 61 nulliparous pregnant women and found no consistent relationship between levator ani muscle dimensions and delivery mode, although they observed an inverse correlation between the urogenital hiatus area, particularly during pelvic floor contraction, and the duration of the second stage of labor [31]. Similarly, Youssef et al. reported no differences in hiatus dimensions between women who had a cesarean section and those who delivered vaginally but they did find an association between a larger hiatus and a longer second stage of labor [32].

Unlike previous findings, our study did not find a significant association between hiatus distensibility during the Valsalva maneuver and labor type or duration. This contrasts with Brunelli et al., who reported a significant association between hiatus distensibility under Valsalva and the duration of the second stage of labor [33].

Additionally, while our study did not find an association between hiatus dimensions and delivery mode, other studies have reported such a correlation [34,35]. For instance, a case–control study with 40 patients observed that those who had a cesarean section had a smaller hiatus area at rest, during levator ani muscle contraction, and under Valsalva [21]. In 2022, Bjerkholt et al. found that both 2D anteroposterior diameter and 3D hiatal area at rest were smaller in women who had an operative delivery compared to those who had a spontaneous vaginal delivery. They also observed an inverse correlation between the second stage of labor and the anteroposterior diameter as well as with the hiatus area at rest and during contraction [19].

In our study, we observed an overall rate of levator ani muscle avulsion of 38.8%. This is a higher rate compared to that reported in previous studies (16% to 36%) [36,37,38,39]. This discrepancy may be partly explained by differences in the study populations: while most existing studies focus exclusively on nulliparous women, our cohort included both nulliparous and multiparous participants, who may have an already damaged pelvic floor. To the best of our knowledge, no other studies have evaluated the impact of a structured prenatal exercise program on pelvic floor muscle trauma resulting from vaginal delivery. Levator ani muscle avulsion is a relatively common obstetric injury associated with vaginal birth and has been linked to an increased risk of long-term pelvic floor dysfunction, including pelvic organ prolapse (POP) and urinary incontinence [40]. Although pelvic floor muscle training in the postpartum period is widely recommended, its effectiveness in facilitating recovery from levator ani muscle avulsion remains controversial. In a clinical trial conducted in 2022 involving 92 primiparous women diagnosed with levator ani muscle avulsion, face-to-face physiotherapy was found to be effective in reducing the hiatal area between six and nine months postpartum [41]. Conversely, another randomized trial in the same year, which included 175 primiparous women with complete levator ani muscle avulsion, reported that early postpartum pelvic floor muscle training did not improve recovery outcomes when compared to women who did not perform the exercises [42]. A supervised exercise program during pregnancy may help prevent levator ani muscle injuries associated with vaginal childbirth. Its potential long-term effects on the development of pelvic floor disorders, such as POP, should be further investigated in randomized controlled trials.

### 4.3. Study Strengths and Limitations

The main strength of our study is its randomized controlled design, which reduces selection bias and enhances internal validity. Additionally, the intervention was homogeneous among all participants, following a standardized and structured protocol under international recommendations [24], thus supporting reproducibility and comparability.

However, our study also has some limitations. The most significant is the small sample size, which may limit statistical power and explain the absence of differences between study groups, but this was a secondary analysis and therefore not powered to detect significant difference but to elaborate new research hypotheses. This limitation also precluded subgroup analyses (e.g., by parity), even though parity is a known confounder in pelvic floor measurements. Finally, we did not perform baseline pelvic floor measurements before randomization and therefore it was not possible to assess the longitudinal effect of the exercise program in the pelvic floor adaptation. This limitation reduces internal validity, as any differences observed between groups in late pregnancy or postpartum may partly reflect preexisting anatomical variability rather than exercise-induced changes. Future trials should incorporate baseline ultrasound assessment, allowing investigators to evaluate the longitudinal changes produced by the exercise intervention.

### 4.4. Clinical Implications

Our findings suggest that participating in a supervised exercise program during pregnancy does not adversely alter pelvic floor morphology or influence the type or duration of delivery. These results support the safety of prenatal exercise and reinforcing the recommendation of supervised prenatal exercise programs as part of routine prenatal care. The results suggest a potential protective effect of prenatal exercise in reducing the incidence of pelvic floor trauma—specifically levator ani muscle avulsion—which may contribute to preserving pelvic floor integrity in the postpartum period. However, the lower pelvic floor contractility observed in the exercise group raises questions about the potential changes in muscle function. Further investigation is warranted to determine whether different types or intensities of exercise produce distinct effects on pelvic floor dynamics, and whether such changes impact postpartum recovery or long-term pelvic floor health.

## 5. Conclusions

A supervised exercise program during pregnancy appears to modify pelvic floor morphology and function, reducing the incidence of levator ani muscle avulsion without affecting the type or duration of delivery. These findings support the safety and potential protective role of prenatal exercise in maintaining pelvic floor integrity. Future studies should investigate the long-term clinical implications and whether specific exercise modalities yield greater benefit for pelvic floor health and postpartum recovery.

## Figures and Tables

**Table 1:** Maternal and pregnancy characteristics of the study population.

	Control (N = 41)	Exercise (N = 37)
Age (years)	33.9 (4.2)	32.6 (4.2)
Body mass index (kg/m)	24.5 (4.4)	24.1 (4.2)
Conception method		
Spontaneous	39 (97.5%)	32 (88.9%)
Assisted	1 (2.5%)	4 (11.1%)
Ethnicity		
Caucasian	40 (97.6%)	35 (94.6%)
Latin-American	1 (2.4%)	2 (5.4%)
Parity		
Nulliparous	25 (61.0%)	26 (70.3%)
Multiparous	16 (39.0%)	11 (29.7%)
Smoking before pregnancy		
No	32 (78.0%)	30 (81.1%)
Yes	9 (22.0%)	7 (18.9%)
Alcohol before pregnancy		
No	22 (53.7%)	20 (54.1%)
Yes	19 (46.3%)	17 (45.9%)
Previous preterm delivery		
No	38 (97.4%)	36 (97.3%)
Yes	1 (2.6%)	1 (2.7%)
Previous cesarean delivery		
No	37 (90.2%)	35 (97.2%)
Yes	4 (9.8%)	1 (2.8%)

Continuous variables are presented as Mean (SD) and categorical variables as n (%). *p*-values are from a Mann–Whitney U or Fisher’s exact test as appropriate.

**Table 2:** Pelvic floor measurements and dynamic changes in maximal contraction and Valsalva maneuver at the second trimester of pregnancy.

	Control (N = 41)	Exercise (N = 37)	*p*-Value
Anteroposterior hiatal diameter at rest (mm)	4.91 (0.76)	4.60 (0.62)	0.049
Latero-lateral hiatal diameter at rest (mm)	3.36 (0.52)	3.37 (0.67)	1
Hiatal area at rest (mm)	12.18 (2.77)	11.16 (1.92)	0.180
Pubovisceral muscle thickness (mm)	1.02 (0.33)	1.11 (0.92)	0.387
Avulsion			0.417
No	36 (90.0%)	32 (86.5%)	
Yes	3 (7.5%)	5 (13.5%)	
Anteroposterior hiatal diameter at contraction (mm)	4.13 (0.62)	3.97 (0.64)	0.315
Later o-lateral hiatal diameter at contraction (mm)	3.06 (0.48)	3.17 (0.48)	0.270
Hiatal area at contraction (mm)	9.53 (2.04)	9.72 (2.06)	0.771
Pubovisceral muscle thickness at contraction (mm)	0.94 (0.19)	0.91 (0.20)	0.478
Anteroposterior hiatal diameter at Valsalva (mm)	4.99 (0.77)	4.93 (0.92)	0.543
Latero-lateral hiatal diameter at Valsalva (mm)	3.49 (0.54)	3.50 (0.63)	0.921
Hiatal area at Valsalva (mm)	13.23 (3.22)	12.96 (3.85)	0.357
Pubovisceral muscle thickness at Valsalva (mm)	0.97 (0.18)	0.98 (0.22)	0.954
Anteroposterior hiatal diameter contractility (%)	14.78 (14.16)	13.50 (11.24)	0.177
Latero-lateral hiatal diameter contractility (%)	8.54 (9.48)	4.04 (11.68)	0.012
Hiatal area contractility (%)	20.15 (16.90)	12.55 (13.18)	0.020
Anteroposterior hiatal diameter distensibility (%)	3.21 (13.48)	8.36 (19.33)	0.478
Latero-lateral hiatal diameter distensibility (%)	4.41 (12.41)	4.89 (14.30)	0.955
Hiatal area distensibility (%)	11.49 (23.12)	17.75 (35.55)	0.559

Continuous variables are presented as Mean (SD) and categorical variables as n (%). *p*-values are from a Mann–Whitney U or Fisher’s exact test as appropriate.

**Table 3:** Labor outcomes.

	Control (N = 41)	Exercise (N = 37)	*p*-Value
Gestational age at delivery (weeks)	39.2 (1.50)	39.3 (1.53)	0.766
Labor onset			0.291
Spontaneous labor onset	22 (53.7%)	13 (36.1%)	
Induction of labor	16 (39.0%)	18 (50.0%)	
Planned cesarean section	3 (7.3%)	5 (13.9%)	
Use of oxytocin	19 (46.3%)	18 (48.6%)	1
Use of epidural	33 (84.6%)	26 (72.2%)	0.385
Mode of delivery			0.692
Cesarean section	5 (12.5%)	6 (16.7%)	
Spontaneous delivery	29 (72.5%)	23 (63.9%)	
Instrumental delivery	6 (15.0%)	7 (19.4%)	
Episiotomy			1
No	34 (97.1%)	30 (96.8%)	
Yes	1 (2.9%)	1 (3.2%)	
Perineal tear			0.130
No	12 (30.0%)	10 (27.8%)	
First degree	14 (35.0%)	5 (13.9%)	
Second degree	9 (22.5%)	13 (36.1%)	
Third degree	0 (0%)	2 (5.6%)	
Duration of the first stage (minutes)	252 (217)	300 (251)	0.605
Duration of the second stage (minutes)	98.1 (86.3)	102 (97.2)	0.949
Birthweight (g)	3150 (448)	3250 (406)	0.571

Continuous variables are presented as Mean (SD) and categorical variables as n (%). *p*-values are from a Mann–Whitney U or Fisher’s exact test as appropriate.

**Table 4:** Pelvic floor measurements and dynamic changes in maximal contraction and Valsalva maneuver at postpartum period.

	Control (N = 30)	Exercise (N = 24)	*p*-Value
Anteroposterior hiatal diameter at rest (mm)	4.79 (0.75)	4.72 (0.63)	0.787
Latero-lateral hiatal diameter at rest (mm)	3.26 (0.49)	3.34 (0.44)	0.632
Hiatal area at rest (mm)	11.54 (2.33)	11.51 (2.05)	0.855
Pubovisceral muscle thickness (mm)	0.92 (0.17)	0.93 (0.22)	0.841
Avulsion			0.009
No	14 (46.7%)	19 (79.2%)	
Yes	16 (53.3%)	5 (20.8%)	
Anteroposterior hiatal diameter at contraction (mm)	4.00 (0.55)	4.00 (0.64)	0.470
Latero-lateral hiatal diameter at contraction (mm)	3.14 (0.58)	3.04 (0.42)	0.741
Hiatal area at contraction (mm)	9.54 (2.43)	9.42 (1.88)	0.632
Pubovisceral muscle thickness at contraction (mm)	1.12 (1.47)	0.88 (0.16)	0.663
Anteroposterior hiatal diameter at Valsalva (mm)	5.32 (0.72)	5.01 (0.88)	0.172
Latero-lateral hiatal diameter at Valsalva (mm)	3.86 (0.72)	3.58 (0.50)	0.156
Hiatal area at Valsalva (mm)	15.61 (4.11)	13.57 (3.73)	0.052
Pubovisceral muscle thickness at Valsalva (mm)	1.02 (0.18)	0.94 (0.16)	0.220
Anteroposterior hiatal diameter contractility (%)	15.78 (9.96)	14.66 (11.84)	0.295
Latero-lateral hiatal diameter contractility (%)	3.56 (13.06)	8.47 (11.08)	0.295
Hiatal area contractility (%)	16.17 (17.52)	16.92 (15.68)	0.623
Anteroposterior hiatal diameter distensibility (%)	12.22 (15.51)	6.40 (13.42)	0.215
Latero-lateral hiatal diameter distensibility (%)	18.89 (16.86)	7.64 (10.39)	0.011
Hiatal area distensibility (%)	36.64 (32.36)	18.01 (23.46)	0.048

Continuous variables are presented as Mean (SD) and categorical variables as n (%). *p*-values are from a Mann–Whitney U or Fisher’s exact test as appropriate.

## Data Availability

The raw data supporting the conclusions of this article will be made available by the authors on request.

## Supplementary Materials

The following supporting information can be downloaded at: https://www.mdpi.com/article/10.3390/diagnostics15151853/s1, Table S1: Logistic regression models for mode of delivery; Table S2: Linear regression models for duration of the second stage of delivery; Table S3: CONSORT 2025 checklist.

## Abbreviations

The following abbreviations are used in this manuscript:

RCT	Randomized controlled clinical trial
ACOG	American College of Obstetricians and Gynecologists (ACOG)
BMI	Body mass index
TUI	Tomographic ultrasound imaging
SD	Standard deviation
POP	Pelvic organ prolapse
